# Long-Term Outcomes and Complications of Trabeculectomy for Secondary Glaucoma in Patients with Familial Amyloidotic Polyneuropathy

**DOI:** 10.1371/journal.pone.0096324

**Published:** 2014-05-06

**Authors:** Takahiro Kawaji, Toshihiro Inoue, Ryuhei Hara, Daisuke Eiki, Yukio Ando, Hidenobu Tanihara

**Affiliations:** 1 Department of Ophthalmology, Faculty of Life Sciences, Kumamoto University, Kumamoto, Japan; 2 Department of Neurology, Faculty of Life Sciences, Kumamoto University, Kumamoto, Japan; Bascom Palmer Eye Institute, University of Miami School of Medicine, United States of America

## Abstract

**Objective:**

Secondary glaucoma is a serious complication in patients with transthyretin (TTR)-related familial amyloidotic polyneuropathy (FAP). We assessed the long-term outcomes and complications of trabeculectomy with mitomycin C (MMC) for secondary glaucoma associated with FAP.

**Methods:**

Medical case records of Kumamoto University Hospital were retrospectively reviewed. Twenty-one eyes of 13 patients (10 with FAP ATTR Val30Met; 3 with FAP ATTR Tyr114Cys) underwent trabeculectomy with MMC and follow-up of at least 2 years. The primary outcome measure was Kaplan-Meier survival, with failure of this treatment being defined as an intraocular pressure (IOP) of ≤5 mm Hg or ≥22 mm Hg on two consecutive visits or as additional operations needed to reduce IOP. Secondary outcome measures included complications, bleb characteristics, and additional postoperative interventions required.

**Results:**

The mean postoperative follow-up period was 5.7 years (range, 2.2–12.7 years). Kaplan-Meier analysis indicated probabilities of success of 0.76, 0.67, and 0.53 at 1, 2, and 3 years after operation, respectively. Significant complications included ocular decompression retinopathy in 7 eyes (33%) and bleb encapsulation in 10 eyes (48%). Twelve eyes (57%) needed additional surgery, such as bleb revision or trabeculectomy with MMC, to reduce IOP.

**Conclusions:**

Trabeculectomy with MMC may not be optimal for patients with FAP-related glaucoma and may have several significant complications.

## Introduction

Transthyretin (TTR)-related familial amyloidotic polyneuropathy (FAP), which is characterized by systemic accumulation of mutant amyloidogenic TTR (ATTR) in organs and peripheral nerves, is a fatal amyloidosis that is inherited in an autosomal dominant fashion [1]. Of more than 100 ATTR mutations, FAP ATTR Val30Met is highly prevalent in Portugal, Sweden, Japan, and other countries. In Portugal and Japan, the penetrance is higher and symptoms typically develop before the age of 40. In contrast, in Sweden, the penetrance is very low and symptoms usually develop after the age of 50 [1]. Patients with TTR-related FAP commonly have ocular manifestations, especially vitreous opacity and glaucoma, which are troublesome and can restrict the daily lives of patients; these ocular involvements occur more frequently during the course of the illness [2]–[4].

We previously reported on the short-term outcomes of surgical treatment of patients with secondary glaucoma associated with TTR-related FAP and the clinical features of these patients [5]. Glaucoma occurred in 12 (24%) of 49 patients; amyloid deposition at the pupillary border, a pupillary border with irregularity, and vitreous opacity were strongly related to the occurrence of glaucoma; and intraocular pressure (IOP) was well controlled during the short follow-up period (mean, 1.2 years; range, 0.2–6.5 years). Also, we suggested that trabeculectomy with mitomycin C (MMC) may be the most promising treatment modality. However, during a longer follow-up period, certain patients required additional operations, and several specific complications developed after surgery.

The study described here therefore continued our evaluation of the efficacy of trabeculectomy with MMC in patients with glaucoma secondary to TTR-related FAP.

## Methods

### Subjects and Data Collection

We retrospectively reviewed the medical records of patients with secondary glaucoma associated with FAP who underwent trabeculectomy with MMC at the Department of Ophthalmology, Kumamoto University Hospital, between 1987 and 2011. Eyes that had had a postoperative follow-up of less than 2 years were excluded. All records were evaluated for clinical signs of FAP, such as polyneuropathy, autonomic dysfunction, and visual disturbance, and later had a definitive diagnosis of FAP on the basis of amyloid deposits found in biopsy samples and genetic investigations. The age at onset of FAP was defined as the time at which the patient first complained of the typical symptoms just mentioned. Visual acuity was measured via standard Landolt C charts; values were converted to letter scores by using the Early Treatment Diabetic Retinopathy Study chart. The Institutional Review Board of Kumamoto University approved this retrospective review and analysis of patient data. Patient records/information was anonymized and de-identified prior to analysis.

### Outcome Measures

The main outcome measure was success or failure of trabeculectomy with MMC as determined according to Kaplan-Meier analysis. Surgical success was defined as an IOP value of ≥6 mm Hg and ≤21 mm Hg with or without antiglaucoma medication. Failure was defined as an IOP value, on two consecutive visits, of ≤5 mm Hg or ≥22 mm Hg with or without antiglaucoma medication or as a need for additional glaucoma operations (excluding laser suture lysis) to reduce the IOP. Secondary outcome measures included complications, bleb characteristics, and postoperative interventions required. IOP data obtained within 2 months after trabeculectomy were excluded because of IOP fluctuations.

### Surgical Procedures

We performed trabeculectomy with MMC according to a modified technique of Cairns: a limbus-based conjunctival flap was used until May 31, 2005, whereas a fornix-based conjunctival flap was used after June 1, 2005, followed by a half-layer scleral flap. We changed to conjunctival flap incisions as this made the surgery easier; the outcomes were similar in our general practice. Small pieces of MMC-soaked sponge (0.4 mg/ml) were applied to exposed tissues, including the Tenon capsule and the posterior surface of the conjunctiva, adjacent episcleral tissue, and scleral flap, for 3–4 min. After removal of all sponges, wounds were irrigated with 200 ml of balanced salt solution. A trabecular block was excised, and peripheral iridectomy was then performed. Closure of the scleral flap and conjunctiva was achieved with 10-0 monofilament nylon sutures. Postoperatively, all patients received the topical regimen of 0.1% betamethasone, 1% atropine sulfate, and 0.5% levofloxacin for 6–12 weeks [6]. Laser suture lysis was performed if the IOP and condition of bleb formation necessitated it. For eyes that required operations to reduce the IOP after the initial trabeculectomy with MMC, we used alternatives such as subconjunctival needle revision [7], repeated trabeculectomy with MMC, and reopening of the scleral flap with MMC [8]–[9].

### Data Analysis

Kaplan-Meier survival analysis was used to determine the cumulative probability of success. We avoided comparing the results for both types of FAP because of low numbers of each type.

## Results

### Clinical Characteristics

Table 1 provides demographic and clinical characteristics of glaucoma patients due to FAP. Of 43 glaucomatous eyes, 27 (63%) had glaucoma surgery. The main indication for primary or additional glaucoma operations was inadequate IOP control despite use of maximal dosages of antiglaucoma medications; the surgical alternatives were selected at the clinician’s discretion. Gonioscopic analysis revealed open angles with mild or heavy pigmentation in all eyes except two, which showed neovascularisation before surgery. These operations included trabeculectomy with MMC for 25 eyes; trabeculectomy without MMC for 1 eye; cyclodestructive procedures (performed at another hospital) for 1 eye; and trabeculotomy with sinusotomy for 1 eye, with a follow-up of only 4 months because of a poor systemic condition. Four of 25 eyes that had had trabeculectomy with MMC were excluded because the follow-up period was less than 2 years; therefore, 21 eyes were enrolled in a subsequent analysis. The mean follow-up period after trabeculectomy with MMC was 5.7 years (range, 2.2–12.7 years). Mean preoperative IOP and mean visual acuity values were 35.2 mm Hg and 78.2 letters, respectively, which changed to 20.4 mm Hg and 44.8 letters, respectively, at the last follow-up visit.

**Table 1:** Demographic and Clinical Characteristics of Patients with Secondary Glaucoma due to FAP.

Characteristic	Val30Met	Tyr114Cys	Total
No. of eyes (patients)	33 (21)	10 (6)	43 (27)
Sex (male/female), n	8/13	1/5	9/18
Age at onset of FAP, y (range)	40.1 (28–73)	37.5 (32–43)	39.5 (28–73)
Age at onset of glaucoma, y (range)	49.5 (44–77)	45.5 (39–54)	48.5 (39–77)
Time from onset of FAP to onset of glaucoma, y (range)	10.4 (4–12)	7.8 (4–10)	8.6 (4–12)
No. (%) of eyes with neovascular glaucoma during follow-up	0 (0%)	8 (80%)	8 (19%)
No. (%) of patients who had LT	14 (67%)	3 (50%)	17 (59%)
No. of eyes (patients) that had trabeculectomy with MMC and at least 2-year follow-up	16 (10)	5 (3)	21 (13)
Age at surgery, y (range)	55.3 (42–79)	48.0 (39–50)	52.6 (39–79)
Follow-up period after surgery, y (range)	5.4 (2.2–12.7)	6.3 (2.4–9.2)	5.7 (2.2–12.7)
Prior surgery, no. (%) of eyes			
Vitrectomy	8 (50%)	5 (100%)	13 (62%)
Cataract surgery	8 (50%)	5 (100%)	13 (62%)
Glaucoma surgery			
Trabeculotomy with sinusotomy	1 (6%)	0 (0%)	1 (5%)
NPT with MMC	1 (6%)	1 (20%)	2 (10%)
IOP, mmHg (range)			
Preoperative	34.8 (24–42)	36.5 (30–43)	35.2 (24–43)
Last follow-up	16.0 (8–29)	34.6 (20–52)	20.4 (12–52)
Visual acuity, letters (range)			
Preoperative	83.8 (58–94)	60.6 (15–89)	78.2 (15–94)
Last follow-up	57.8 (0–89)	3.0 (0–15)	44.8 (0–89)

FAP = familial amyloidotic polyneuropathy; IOP = intraocular pressure; LT = liver transplantation; MMC = mitomycin C; NPT = nonpenetrating trabeculectomy.

### Outcomes

**Figure 1:**
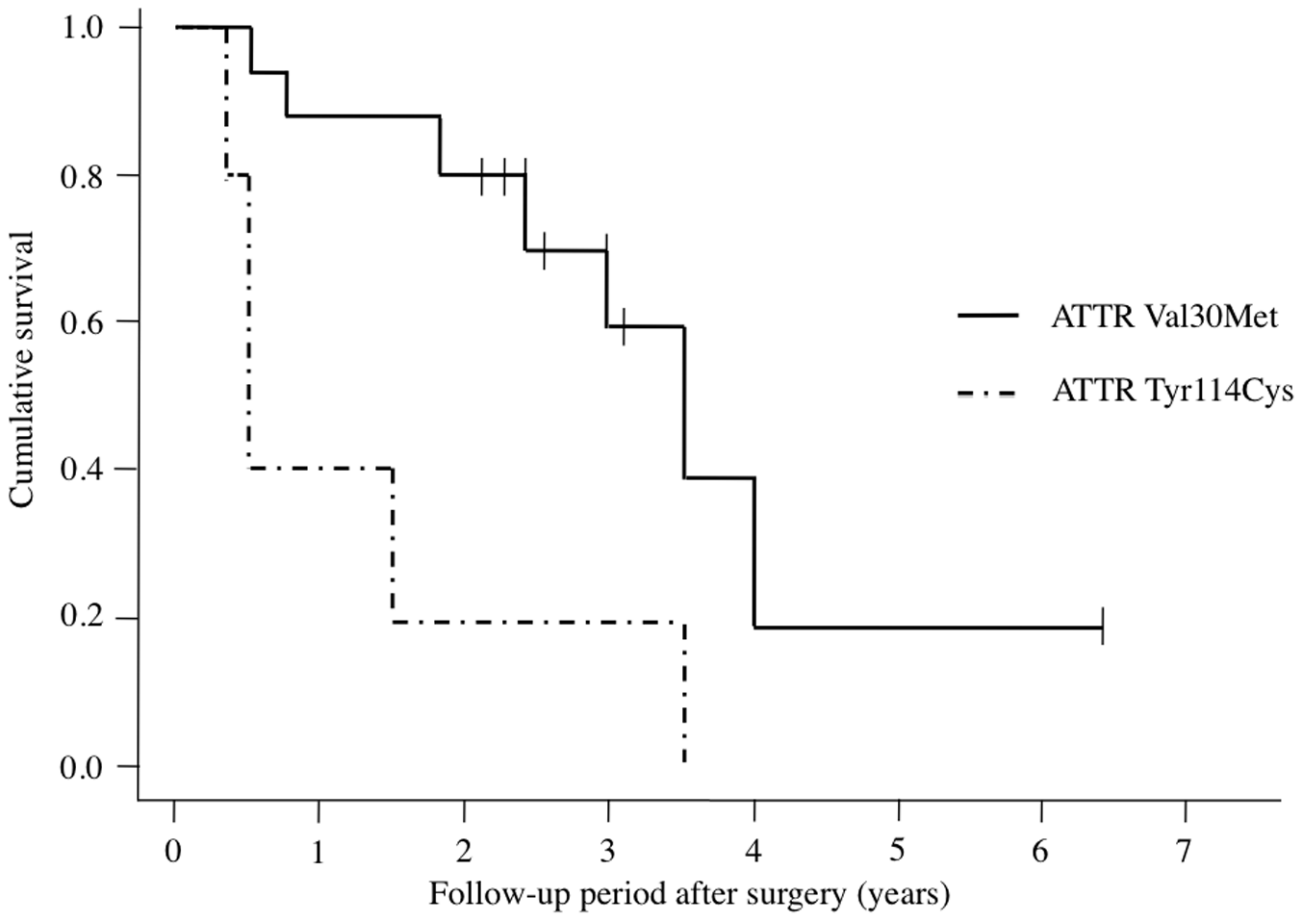
Kaplan-Meier survival curve for patients treated with trabeculectomy with mitomycin C. Treatment failure was defined as an intraocular pressure (IOP) value of ≤5 mm Hg or ≥22 mm Hg on at least two consecutive visits or as additional glaucoma operations (excluding laser suture lysis) required to reduce the IOP. The small vertical dashes along the curves represent times of censored observations.

Figure 1 presents results of Kaplan-Meier survival analysis for 21 eyes that underwent trabeculectomy with MMC. According to treatment success criteria, cumulative survival values at 1 year were 0.76 for both groups, 0.88 for patients with FAP ATTR Val30Met, and 0.40 for patients with FAP ATTR Tyr114Cys. These respective values were 0.67, 0.81, and 0.20 at 2 years; 0.53, 0.63, and 0.20 at 3 years; 0.11, 0.17, and 0 at 4 years. Eight eyes were censored because regular follow-up ended. Eight of 16 eyes (50%) of patients with FAP ATTR Val30Met and all eyes (100%) of patients with FAP ATTR Tyr114Cys were classified as surgical failures.

### Interventions and Complications

Table 2 lists reasons for treatment failure, as well as complications, bleb characteristics, and additional operations. Three of five eyes (60%) of patients with FAP ATTR Tyr114Cys had no light perception because of subsequent neovascular glaucoma, which may have been caused by retinal ischemia induced by ocular amyloid angiopathy. Significant complications during the follow-up period included ocular decompression retinopathy (7 eyes, 33%) and bleb encapsulation (10 eyes, 48%).

**Table 2:** Summary of Interventions and Complications.

Outcome	Val30Met	Tyr114Cys	Total
	(n = 16 eyes)	(n = 5 eyes)	(n = 21 eyes)
High IOP	8 (50%)	5 (100%)	13 (62%)
Loss of light perception	1 (6%)	3 (60%)	3 (14%)
Complications			
Bleb leakage	1 (6%)	0 (0%)	1 (5%)
Choroidal detachment	2 (13%)	0 (0%)	2 (10%)
Ocular decompression retinopathy	3 (19%)	4 (80%)	7 (33%)
Bleb characteristics			
Avascularity	2 (13%)	1 (20%)	3 (14%)
Delayed bleb leaks (Seidel’s test)	2 (13%)	0 (0%)	2 (10%)
Encapsulation	7 (44%)	3 (60%)	10 (48%)
Additional surgical procedures*			
Bleb needling	2 (13%)	0 (0%)	2 (10%)
Bleb revision for leakage	1 (6%)	0 (0%)	1 (5%)
Bleb revision (Reopening of the scleral flap with MMC)	4 (25%)	2 (40%)	6 (29%)
Trabeculectomy with MMC	5 (31%)	0 (0%)	5 (24%)
Cyclodestructive procedure	0 (0%)	1 (20%)	1 (5%)

IOP = intraocular pressure; MMC = mitomycin C.

*Some eyes had more than 1 procedure.

Twelve eyes (57%) of 8 patients required additional procedures to reduce IOP. Four eyes required more than one additional treatment. Six eyes had the scleral flap reopened via MMC; five eyes had another trabeculectomy with MMC. The mean time from initial trabeculectomy to additional surgery was 1.9 years (range, 0.2–6.0 years).

## Discussion

Before the utilization of liver transplantation, early-onset FAP (before the age of 50) was fatal, with an expected survival of about 10 years from disease onset [1]. Because the liver is the main site of synthesis of ATTR found in serum, liver transplantation has been thought to be a promising approach for halting progression of neurological complications in patients with TTR-related FAP [10]. However, ocular complications have continued and indeed worsened after liver transplantation [5], [11], because ocular tissues—the retinal and ciliary pigment epithelia—also synthesised ATTR [12], [13]. In continuing investigations of these ocular complications and their treatment, we previously evaluated 17 glaucomatous eyes of 10 patients and 15 surgically treated eyes of 9 patients, but these numbers increased to 43 eyes of 27 patients and 21 eyes of 13 patients, respectively, in the present study.

Glaucoma is the most serious ocular manifestation of FAP and can cause severe visual disturbances. Previously reported evidence led to the common belief that ocular amyloid deposition may result in obstruction of the aqueous outflow route and/or perivascular amyloid deposition in conjunctival and episcleral tissues, which may contribute to elevated episcleral venous pressure and thereby cause glaucoma [5], [14], [15]. Our previous study demonstrated that the findings of amyloid deposition at the pupillary border, a pupillary border with irregularity, and vitreous opacity seemed to be reliable for predicting glaucoma onset [5]. Also, trabeculectomy with MMC appeared to be the most promising treatment modality available to us on the basis of our previous short-term results [5]. However, our present study providing longer term follow-up results revealed that the effect of trabeculectomy with MMC seemed to be limited on FAP-associated glaucoma. The cumulative probability of treatment success in our study was 0.76 at 1 year, 0.67 at 2 years, 0.53 at 3 years and 0.11 at 4 years. Approximately half of the trabeculectomized eyes needed additional surgical procedures within 2 years after surgery. We recently reported on the outcomes for 101 patients who underwent trabeculectomy with MMC for neovascular glaucoma, which is particularly difficult to manage; the probability of treatment success at 1, 2, and 5 years after surgery was 0.63, 0.58, and 0.52, respectively [6]. Thus, glaucoma associated with FAP appears to present serious management problems.

Bleb encapsulation, whose features include a dome-shaped, tense, opalescent, thick-walled bleb with vascular engorgement of overlying conjunctiva, is a relatively uncommon complication of filtering surgery. The occurrence of bleb encapsulation in different types of glaucoma is reportedly between 2.5% and 29%, depending on the surgical technique used [16]–[18]. Various risk factors, including male sex, prior use of topical medications, argon laser trabeculoplasty, glaucoma diagnosis, and surgery involving the conjunctiva, have been associated with development of this complication [16], [17]. In our study, we found 10 eyes (48%) with bleb encapsulation, accompanied by an elevated IOP. Because 7 of these eyes had had vitrectomy for vitreous opacity before the trabeculectomy, vitrectomy may be related to the development of bleb encapsulation. Ophir proposed that comparatively mild triggers of fibroblast activation, such as prolonged preoperative use of antiglaucoma medication and ocular trauma, may promote bleb encapsulation, whereas stronger stimuli such as previous intraocular surgery promote bleb scarring [18]. Other researchers reported that, in patients with TTR-related FAP, TTR aggregates that induce abnormal, sustained activation of extracellular signal-regulated kinases 1/2 may result in activation of nuclear transcription factor-κB, up-regulation of proinflammatory cytokines, oxidative stress, and ultimately neurodegeneration [19], [20]. Amyloid was also often deposited in the subepithelial layer and vascular wall of the conjunctiva [14], [21]. Therefore, TTR aggregates in the conjunctiva induce mild fibroblast activation, which may result in bleb encapsulation. Further study is needed to elucidate whether amyloid deposition affects development of bleb encapsulation.

In the present study, almost half of trabeculectomized eyes needed additional surgical interventions because trabeculectomy failed within 2 years after the operation. For the most recent cases, we utilized MMC to reopen the scleral flap, because most patients were relatively young and because of possible advantages of reopening the failed filter: reopening ensures superior filtration and preserves the superior conjunctiva for possible future surgery. However, this procedure, as well as other methods such as repeated trabeculectomy and needle revision, had limited efficacy. Although glaucoma drainage implants are now regarded as promising therapeutic options in types of glaucoma that are difficult to manage such as neovascular glaucoma, drainage implant procedures had not been performed at our institution yet, because these devices had not been approved for clinical use in Japan until 2012. After their approval, further study will be needed to determine the effects of drainage implants on FAP-associated glaucoma. We recently reported results of a pilot trial in which panretinal laser photocoagulation prevented progression of amyloid deposition in the vitreous and on the retinal surface [22]. However, the effect of laser photocoagulation on glaucoma progression is still unknown, and additional study is warranted.

In our study described here, we detected 7 eyes (33%) with ocular decompression retinopathy, which is a rare complication characterized by scattered retinal hemorrhages occurring immediately after uncomplicated filtering surgery [23], [24]. Fechtner *et al* hypothesized that one possible cause of this complication is a loss of retinal vessel autoregulation, which overwhelms the ability of the vessels to respond to IOP changes and results in retinal hemorrhages [23]. Our present FAP patients manifested autonomic dysfunction as one of their systemic symptoms, which increased their susceptibility to such a phenomenon. The hemorrhages resolved spontaneously in all patients, and only one patient showed decreased visual acuity. In addition, 8 eyes (80%) of 5 patients with FAP ATTR Tyr114Cys developed neovascular glaucoma later, possibly caused by retinal ischemia. We previously reported the occurrence of ocular amyloid angiopathy in patients with FAP ATTR Tyr114Cys, in whom vascular amyloid deposition caused a steno-occlusive vascular disorder that resulted in ischemic changes [25]. Patients with FAP ATTR Tyr114Cys have a susceptibility to retinal ischemic changes, so they have a poor prognosis compared with patients with FAP ATTR Val30Met. Thus, these complications—ocular decompression retinopathy and neovascular glaucoma caused by amyloid angiopathy—appear to be related to the FAP disease process rather than to surgical procedure.

Our study has several limitations. This is a retrospective review of cases performed consecutively. There is no control group for comparison of our results. All patients are Japanese, and the number of patients is small. Another limitation is the use of both eyes in a single patient. However, given the rarity of this disease, we do not feel that it was inappropriate to do so.

In summary, as a consequence of the significantly improved survival of patients with FAP that is achieved by liver transplantation, glaucoma may become a more common serious complication. Our results suggest that trabeculectomy with MMC may not have a sufficient effect on secondary glaucoma in patients with FAP and that this method has several significant complications. Besides larger and long-term follow-up studies, glaucoma drainage implant studies are needed to determine effective therapeutic strategies.
